# Development of an mHealth Solution for Tailored Communication Between Patients With Inflammatory Bowel Disease and Health Care Professionals: Participatory Design Study

**DOI:** 10.2196/69093

**Published:** 2025-10-31

**Authors:** Janni Petersen Fallesen, Marie Louise Krogh, Torben Knudsen, Lisbeth Rosenbek Minet, Jens Kjeldsen, Mette Maria Skjøth

**Affiliations:** 1 Department of Nursing, University College Lillebaelt, Niels Bohrs Alle 1, Odense M, 5230, Denmark, 45 25377733; 2 Department of Medical Gastroenterology, Odense University Hospital, Odense, Denmark; 3 Research Unit of Medical Gastroenterology, University of Southern Denmark, Odense, Denmark; 4 Center Denmark, Fredericia, Denmark; 5 Department of Medical Gastroenterology, Esbjerg, University Hospital of Southern Denmark, Esbjerg, Denmark; 6 Department of Regional Health Research, University of Southern Denmark, Esbjerg, Denmark; 7 Department of Geriatrics, Odense University Hospital, Odense, Denmark; 8 Research Unit of Geriatric, Department of Clinical research, University of Southern Denmark, Odense, Denmark; 9 Center for Innovative Medical Technology (CIMT), Odense University Hospital, Odense, Denmark; 10 Department of Dermatology and Allergy, Odense University Hospital, Odense, Denmark

**Keywords:** mobile health, mHealth, inflammatory bowel disease, app, participatory design, communication

## Abstract

**Background:**

Patients with inflammatory bowel disease (IBD) have periods with flare-ups, including abdominal pain, diarrhea, bloody stools, and systemic symptoms that may negatively influence the patients’ quality of life. Hence, prompt and intensified treatment is often required, and patients need to pay attention to self-management, including easy access to health care professionals. Seeking support is essential in patients’ self-management and beneficial for their quality of life. However, patients may experience difficulties in gaining access to health care professionals by phone or email when needed. Mobile health (mHealth) interventions have been shown to support patients with flexible, timely, and ongoing communication with health care professionals. However, the most prevalent functions of current apps for patients with IBD are tracking disease symptoms and accessing information. In addition, patient and clinician involvement in the design and development of eHealth apps for patients with IBD has been limited, although engaging patients is emphasized as essential for identifying tools and functionalities that they find relevant and effective.

**Objective:**

This study aimed to develop an mHealth solution for patients with IBD using participatory design to support tailored communication between patients and health care professionals.

**Methods:**

Through participatory design, we completed 3 focus groups, 4 mock-up workshops, and 2 prototype tests involving patients, health care professionals, and an IT designer to collaboratively develop a prototype. The iterative process allowed for feedback from all stakeholders to inform the design and development. This approach facilitated ongoing refinement of the prototype until a mutually satisfactory solution was achieved. Data analysis followed the structured phases inherent to participatory design: planning, acting, observing, and reflecting.

**Results:**

A total of 14 patients with IBD aged 18-65 years and 9 health care professionals from 2 outpatient clinics in Denmark contributed to the mHealth design. The analysis generated 6 themes of patients’ suggestions for app content: easy-access messaging, agreement overviews, self-initiated patient-reported outcomes with free text, treatment and blood test notifications, an IBD knowledge base, and self-monitoring via diary and symptom registration. An intervention that reflected users’ needs and requests to support patients’ access to and communication with health care professionals in outpatient clinics was developed. The intervention included messaging, symptom registration, notifications, questionnaires with free-text space, a knowledge base, and an appointment overview.

**Conclusions:**

The participatory design served as a usable approach to designing and developing a tailored mHealth solution for patients with IBD and their health care professionals in an outpatient clinic. On the basis of the iterative design process with mutual learning and democratic voices, the participants had a significant impact on the solution, which reflected users’ needs and resulted in the effective adaptation of the solution to the clinical setting.

## Introduction

### Background

Inflammatory bowel disease (IBD), including ulcerative colitis and Crohn disease, is a chronic, relapsing condition with symptoms such as abdominal pain, diarrhea, and bloody stools [1,2]. These symptoms can impact psychological and social well-being, lowering quality of life [3]. Most patients undergo lifelong treatment and check-ups, with flare-ups often requiring prompt treatment [4,5]. Patients must prioritize self-management, including easy access to health care professionals who may help alleviate living with IBD [3,6,7]. Access to support from health care professionals often takes place by telephone or email [8]. However, patients may experience difficulties in gaining access [3,9,10]. Close communication with health care professionals is essential for supporting patients’ self-management, well-being, and quality of life [6,11]. In addition, handling symptoms consists of adapting diet, medical treatment, stress management, and symptom recognition [6]. Thus, there is a need to improve access to health care professionals and increase self-management among patients with IBD.

eHealth, as defined by World Health Organization [12], involves using information and communication technologies in health care and has become an essential part of modern health care, particularly in the management of chronic conditions such as IBD [13,14]. eHealth is a favored solution among patients with IBD and can potentially improve access to health care professionals [10]. eHealth enables remote self-management [15,16], including symptom monitoring and shared decisions, and has a positive impact on quality of life [16-18]. Mobile health (mHealth) apps are becoming popular, as they contribute to direct care, treatment support, and psychosocial support [10,19,20]. mHealth is defined by the World Health Organization as *use of mobile wireless technologies for public health* [12]. mHealth can support patients with flexible, timely, and ongoing communication with health care professionals, including symptom management, which helps reduce psychological stress [15,21]. However, most IBD apps focus primarily on symptom tracking and accessing information [22].

Previous studies highlighted the importance of involving patients with IBD in designing eHealth technologies to identify tools and functionalities that the patients find relevant and effective [10,23]. However, patient and clinician involvement in developing IBD apps has been limited [10]. IBD app developers have prioritized developing app features while neglecting the importance of making the app user-friendly through customization and stakeholder involvement [22]. In patient involvement, there is evidence for using cocreation methods, including participatory design, to strengthen the implementation and usability of solutions [24,25].

The foundational values and key methods in participatory design are grounded in democratic ideals, emphasizing equal and genuine participation [25]. Co-design, a central method in participatory design, facilitates collaborative engagement between stakeholders and designers, ensuring that outcomes are informed by diverse perspectives and real user needs [25]. Genuine participation includes the opportunity for participants to have a say and mutual learning with stakeholders [25]. The purpose of participatory design is to forecast experiences with future technology before the technology has been developed, and because of the iterative nature of participatory design, each step in the process is planned according to the results and ideas that are created during the preceding activity [26].

### Objectives

This study aimed to develop an mHealth solution for patients with IBD through user involvement to strengthen communication with health care professionals.

## Methods

### Design and Setting

**Figure 1.**
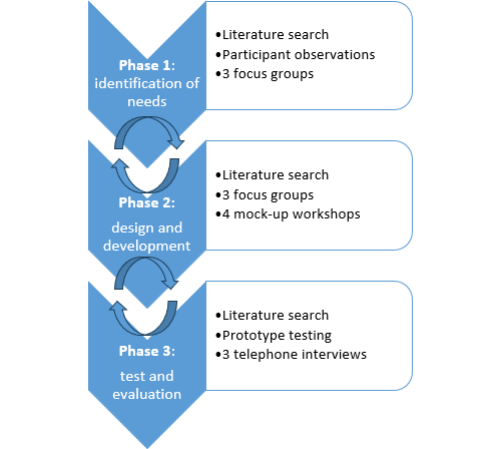
An overview of the activities in the 3 participatory design phases.

Grounded in participatory design [27], this project is structured in 3 phases across 2 studies: study 1 covers phase 1 and study 2 includes phases 2 and 3 (Figure 1). The 3-phase project is based on the Simonsen iterative, user-centered approach [25]. Phase 1 identified user needs through 3 focus groups, each lasting 2 hours, and 111 hours of participant observations, as reported in a previous study [11]. This study presents results from phases 2 and 3, which focused on developing and testing a solution for patients with IBD, informed by phase 1 findings [11]. This study took place at 2 specialized gastroenterology outpatient clinics at Danish university hospitals from March 2019 to December 2019.

In this study, 3 patient focus groups were held to identify app content based on phase 1 findings. Two workshops involving health care professionals addressed patient input from focus groups and workflow integration, while 2 parallel workshops involving patients refined the prototype. In addition, 2 prototype tests were conducted with patients, a nurse, and an IT developer.

**Figure 2.**
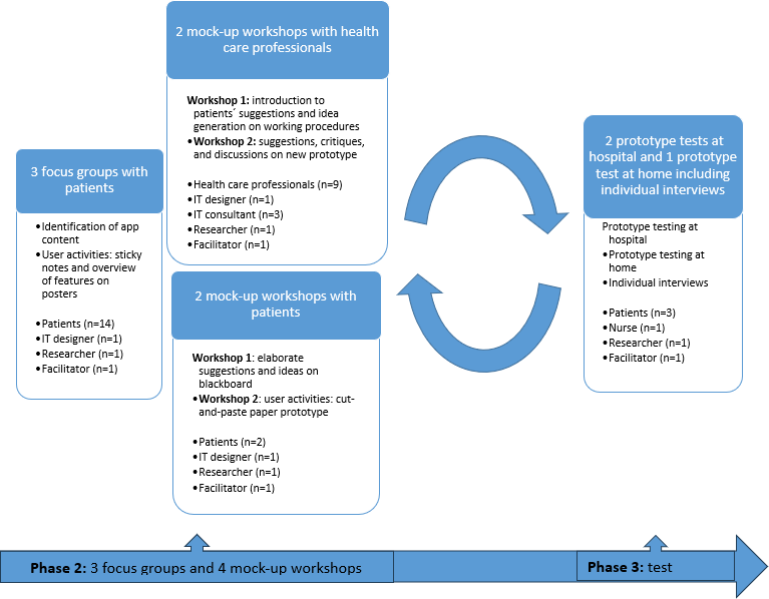
The development and testing process of phases 2 and 3 in the development of the mobile health solution in the participatory design project.

To encourage open dialogue, all focus groups, workshops, and prototype tests were held in neutral hospital settings, away from outpatient clinics. Three patients tested the prototype at home for 3 weeks, followed by telephone interviews after 1 week and 3 weeks (Figure 2). This early testing laid the foundation for funding applications, broader clinical testing, and full implementation.

### Participants

Participants were recruited from 2 specialized gastroenterology outpatient clinics at Danish university hospitals serving regional populations. Using heterogeneous sampling [28], 14 outpatients with IBD were recruited to focus groups based on age, gender, disease, and treatment (including biologics; Table 1). Specialized IBD nurses recruited patients during clinic visits. Inclusion criteria encompassed patients with IBD attending for consultations, medical therapy, or symptom management. Exclusion criteria included non-Danish speakers and those aged <18 years. The 14 focus group patients were invited to mock-up workshops; 2 participated in workshops, and 1 also took part in prototype testing (Table 2).

**Table 1.** Demographic data of patients in focus groups, mock-up workshops, and prototype testing in phases 2 and 3 of a participatory design study at 2 university hospitals in Denmark.

Focus groups, mock-up workshops, and prototype tests	Patients, n (%)	Sex, n (%)		Age (y), n (%)		Disease, n (%)		Ongoing medical treatment, n (%)
		Male	Female	18-35	36-65	Crohn disease	Ulcerative colitis	
Focus group								
Focus group 1	7 (50)	4 (57)	3 (43)	2 (29)	5 (71)	5 (71)	2 (29)	7 (100)
Focus group 2	4 (29)	1 (25)	3 (75)	3 (75)	1 (25)	2 (50)	2 (50)	4 (100)
Focus group 3	3 (21)	1 (33)	2 (67)	0 (0)	3 (100)	2 (67)	1 (33)	2 (67)
Workshop								
Workshop 1	2 (100)	1 (50)	1 (50)	0 (0)	2 (100)	2 (100)	0 (0)	2 (100)
Workshop 2	2 (100)	1 (50)	1 (50)	0 (0)	2 (100)	2 (100)	0 (0)	2 (100)
Prototype test								
Prototype test at hospital 1	2 (100)	1 (50)	1 (50)	0 (0)	2 (100)	2 (100)	0 (0)	2 (100)
Prototype test at hospital 2	1 (100)	0 (0)	1 (100)	0 (0)	1 (100)	0 (0)	1 (100)	1 (100)
Prototype test at home	3 (100)	1 (33)	2 (67)	0 (0)	3 (100)	3 (100)	0 (0)	3 (100)
Individual interviews	3 (100)	1 (33)	2 (67)	0 (0)	3 (100)	3 (100)	0 (0)	3 (100)

**Table 2.** Description of activities and focus group, mock-up workshop, and test participants in phases 2 and 3 in a participatory design study at 2 university hospitals in Denmark.

Activities	Participants in focus groups with patients (n)	Participants in mock-up workshops with health care professionals (n)	Participants in mock-up workshop with patients (n)	Participants in prototype tests at hospital and at home, including individual interviews (n)
First focus group, workshop, and test set	Focus group 1 Patients (7) IT designer (1) Researcher (1) Facilitator (1)	Workshop 1 Nurses (3) Physicians (2) IT designer (1) IT consultants (3) Researcher (1) Facilitator (1)	Workshop 1 Patients (2) IT designer (1) Researcher (1) Facilitator (1)	Test 1 at hospital Patients (2) Nurse (1) IT designer (1) Researcher (1) Facilitator (1)
Second focus group, workshop, and test set	Focus group 2 Patients (4) IT designer (1) Researcher (1) Facilitator (1)	Workshop 2 Nurses (6) Doctors (2) IT designer (1) Researcher (1) Facilitator (1)	Workshop 2 Patients (2) IT designer (1) Researcher (1) Facilitator (1)	Test 2 at hospital Patients (1) Nurse (1) IT designer (1) Researcher (1) Facilitator (1)
Third focus group and test set	Focus group 3 Patients (3) IT designer (1) Researcher (1) Facilitator (1)	Not applicable	Not applicable	Test at home Patients (3) Nurse (1)
Interviews	Not applicable	Not applicable	Not applicable	Individual interviews Patients (3) Researcher (1) Facilitator (1)

Health care professionals with relevant IBD outpatient experience were recruited for 2 workshops via email and personal follow-up. IT consultants from both hospitals were also invited by email due to their expertise in eHealth development.

### Data Collection

#### App Content Identified Through Focus Groups

On the basis of the needs assessment in an explorative qualitative study in phase 1 (Textbox 1) [11], we completed 3 focus groups in phase 2 to explore patients’ perceived needs and app content ideas through spontaneous, exploratory, and lively discussions [29]. All participants were heard with minimal interruptions [29]. An interview guide with open descriptive questions was used to open the conversations, and keywords from findings in phase 1 helped keep discussions focused on their needs [11]. The user-driven focus groups included patients individually noting their top 3 feature priorities on sticky notes [25], presenting and explaining their ideas. Then, the authors discussed them with the group to ensure genuine participation and that all had a say. Participant observations from study 1, health care professionals’ expertise, and current literature also informed elaborate questions to explore potential unmet needs.

Textbox 1. **Phase 1 findings from a participatory design study: end users’ needs in an exploratory qualitative study.**

Themes

- Easy and dependable access to health care professionals
- Predictability of follow-up appointments
- Importance of privacy during patient examinations
- Quality of time spent with health care professionals

**Figure 3.**
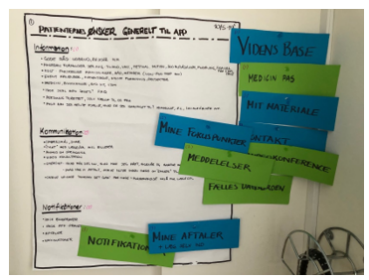
Needs and ideas of patients with inflammatory bowel disease for the content of the mobile health solution.

Patients’ wishes were grouped into categories (eg, information, communication, and notifications) on a poster for clarity (Figure 3). The facilitator and researcher moderated the 2-hour focus groups, with an IT designer present to understand content needs. Data were recorded and transcribed. We chose to integrate the solution into an existing hospital platform by the Region of Southern Denmark, already used in multiple clinical areas to facilitate patient access to health care professionals [30,31]. This secure, General Data Protection Regulation (GDPR)–compliant platform is free for hospital patients and integrated with electronic patient records.

#### Mock-Up Workshops With Health Care Professionals and Patients

We conducted 4 workshops: 2 involving 9 health care professionals, an IT designer, 3 IT consultants, a researcher, and a facilitator; and 2 involving the same 2 patients, an IT designer, a researcher, and a consultant (Table 2). The aim was to strengthen the creative and involving process through genuine participation and mutual learning during the development and design of features that participants found relevant. The IT designer and research team were briefed beforehand on aims, exercises, and plenary discussions in a script. Workshop outputs were documented, including notes, illustrations, discussions, and design suggestions.

#### Mock-Up Workshops With Health Care Professionals

Two workshops were conducted at a neutral place at the 2 university hospitals away from outpatient clinics, each lasting 2 hours. The first workshop introduced health care professionals to patient needs via focus group posters and gathered health care professionals’ input on integrating features with clinic workflows. In the second workshop, the IT developer presented potential solutions based on participant wishes, refined through feedback, leading to a prototype proposal.

#### Mock-Up Workshops With Patients

**Figure 4.**
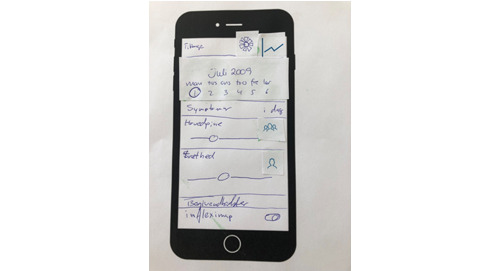
Example of the paper prototype made by patients with inflammatory bowel disease.

**Figure 5.**
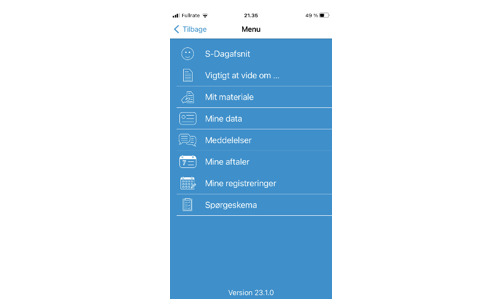
The app prototype with features.

Two workshops were conducted at the IT developer’s office, lasting 3 and 2 hours. The workshops aimed to improve the design and develop the features discussed in the focus groups and workshops with health care professionals. The first workshop involved patients expanding on ideas while the developer visualized them on a blackboard, fostering deeper insight into potential unmet needs. In the second workshop, patients created paper prototypes by assembling features and interface elements (Figure 4). These informed the prototype design alongside earlier discussions (Figure 5).

### Prototype Testing of App in Phase 3

#### Telephone Interviews

After 1 and 3 weeks of home testing by 3 patients, an unstructured telephone interview was conducted to identify needed changes. During testing, patients sent messages to an outpatient nurse, completed questionnaires, recorded symptoms, explored the knowledge base, viewed the appointment schedule, and received notifications. The nurse involved in remote testing also took part in earlier hospital-based prototype tests. Two 1-hour hospital tests were held before home testing. App features were tested individually, with patients using their own smartphones to explore the app intuitively, ask questions, and practice independently before home testing.

#### Analysis

On the basis of the participatory design approach, all data (sound files, posters, illustrations, and sticky notes) from focus groups, workshops, and prototype tests were analyzed iteratively. Two of the authors followed the 4 steps during the iterative processes: plan, act, observe, and reflect [26]. Each workshop was planned based on insights from the previous one, and the next step in the design and development phase was planned based on this mutual learning between participants and developers.

Due to parallel data collection and development of the mHealth solution, timely in-depth data analysis was limited. However, continuity and contextual understanding were preserved by involving the same IT developers throughout design and development. To address the limitations of concurrent development and data collection, a thorough post hoc analysis was conducted to ensure rigorous insights from the collected data.

Focus group data were transcribed and analyzed using the 5-step meaning condensation method by Kvale and Brinkmann [32]. This included the following:

1. Reading the transcribed material for overall impression
2. Identifying units of meaning
3. Reformulating the dominant topics in the units into themes
  - As an example of meaning condensation in this step, the natural meaning unit “I need some kind of messaging feature with nurses. Then it would be easier instead of having to wait in that queue” (Focus group 2, informant 1) was given the theme “messaging.”
4. Posing questions based on the purpose of the study
5. Summarizing findings into key themes

The first author conducted the analysis and identified themes, which were discussed with the full research team. Reporting followed the COREQ (Consolidated Criteria for Reporting Qualitative Studies) guidelines (Checklist 1) [33].

### Ethical Considerations

The study was submitted to the Scientific Ethics Committee of the Region of Southern Denmark, which deemed approval unnecessary according to Danish legislation. The study was recorded in the Region of Southern Denmark’s register of data processing activities (journal 19/47642), and the GDPR was complied with through written informed consent. The app includes 2 consent declarations, one for app use and one for research participation, both accessible before use and revocable at any time. A project description is also available within the app.

## Results

### App Content Identified Through Focus Groups

The analysis identified 6 themes of patients’ app content suggestions: easy-access messaging, agreement overviews, self-initiated patient-reported outcomes with free text, treatment and blood test notifications, an IBD knowledge base, and self-monitoring via diary and symptom registration (Textbox 2).

Textbox 2. **App content identified as important to patients based on focus group findings.**

Desired features

- Messaging
- Self-initiated patient-reported outcome questionnaires
- Overview of agreements
- Treatment and blood sample notifications
- Knowledge base
- Self-monitoring via diary and symptom registration, with the option of sharing the information with health care professionals when needed

The most common suggestion for improving clinical practice was easy access to health care professionals. Patients proposed a messaging feature to avoid disruptive phone queues. One patient shared the following from her sticky note in a focus group:

I need some kind of messaging feature with nurses. Then it would be easier instead of having to wait in that queue.

[Focus group 2, informant 1]

Easy access was perceived as an essential self-management strategy when patients struggled to manage their disease. They preferred messaging for nonurgent issues over phone calls.

Contact with health care professionals often involved dialogue, symptom-related questions, advice for managing the illness, prescriptions, or booking appointments, for example:

A communication module for messaging the clinic, with options to book in-person or phone appointments.

[Focus group 1, informant 3]

Patients also requested to be involved in changes to their health care contacts. They suggested having an overview of their treatment and arranged contacts:

I need a treatment overview showing my current plan, timing, and how it changes with disease activity.

[Focus group 3, informant 3]

Moreover, patients wanted private discussions of their symptoms with health care professionals and suggested including a free-text option in the questionnaire to share details before treatment:

It would be helpful to write in the app the day before, how you feel, so they know in advance, and you don’t have to explain it all in person.

[Focus group 1, informant 2]

The participants had 3 additional wishes for the app: blood test and treatment notifications, a knowledge base and a diary, and a symptom registration tool.

In focus groups, patients expressed a need for blood test and treatment notifications, as they struggled to remember their appointments:

I need reminders for blood tests because I find it hard to remember.

[Focus group 1, informant 3]

Furthermore, the patients stated a need for transparent and trustworthy information in a knowledge base on how to live with IBD:

I need reliable advice, as I’ve found confusing information online; the app should provide clear, basic guidance.

[Focus group 2, informant 1]

They also requested a diary and symptom tracker to monitor flare-ups, with options to share data with health care professionals:

I need a symptom record to accurately recall when symptoms occurred, their duration, and severity for doctor visits.

[Focus group 1, informant 4]

Patients also needed a diary tool to monitor daily activities and diets that might affect their disease:

I can use it to track in periods where I feel changes that could be due to flare-ups, stress, or overexertion, helping identify patterns or external factors affecting my condition. [Focus group 1, informant 2]

### Mock-Up Workshops With Health Care Professionals and Patients

**Figure 6.**
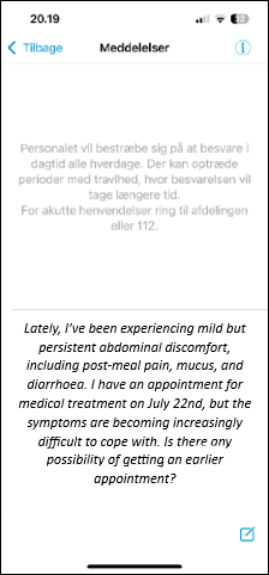
An example of a patient message in the messaging tool that helps patients to obtain easy access to health care professionals.

**Figure 7.**
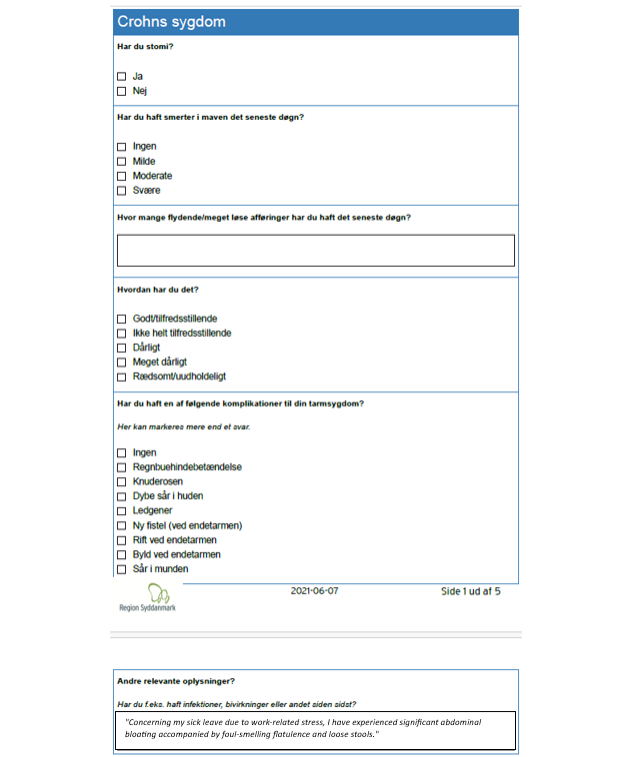
Self-initiated patient-reported outcome questionnaire specific to Harvey-Bradshaw Index, with an example of a patient message to health care professionals in the free-text space.

On the basis of the 3 focus groups, an mHealth solution was developed for patients with IBD in an outpatient setting (Textbox 2). The prototype included asynchronous messaging with nurses, allowing responses within 48 hours, a compromise between patient requests for rapid replies and clinical feasibility (Figure 6). Notifications for blood tests and treatments, sent 5 and 2 days before biological therapy, respectively, were also developed. Patients were given the opportunity to complete symptom questionnaires (Simple Clinical Colitis Activity Index or Harvey-Bradshaw Index) at home and use a free-text space to express how they felt and their privacy preferences for nurse communication (Figure 7).

**Figure 8.**
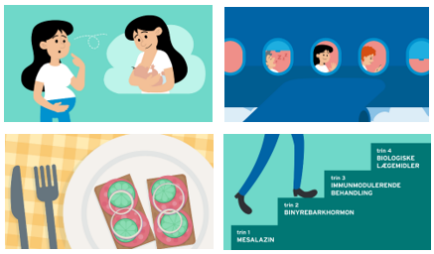
Participants selected figures and illustrations for content in the knowledge base.

**Figure 9.**
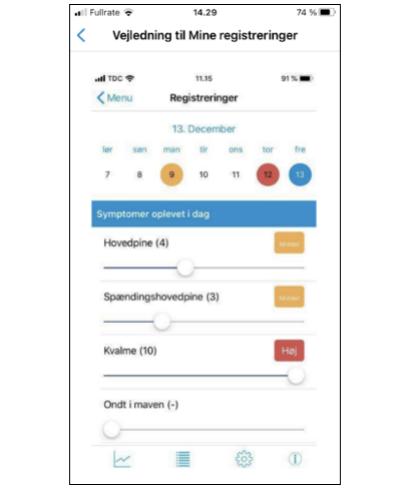
Visualization of the diary and symptom registration tool that patients can use to monitor their own symptoms in daily life, while also having the opportunity to share this information with health care professionals.

Content for the knowledge base, covering medical treatment, pregnancy, vaccines, and travel, was created by 4 nurses and 3 doctors, with input from a physiotherapist and dietician, all based on professional guidelines. A communications consultant advised on patient-friendly text and reviewed the content. Additional links to patient associations and blood sample ordering were included. Participants selected figures, illustrations, and an animated video with a graphic designer (Figure 8). A diary and symptom-tracking feature was developed to allow patients to record the duration and severity of symptoms during flare-ups, with the option to share these records with health care providers (Figure 9).

### Phase 3: Prototype Testing

After 1 and 3 weeks of home testing, unstructured telephone interviews were conducted to identify needed adjustments. Patients found the app easy and safe to use for completing questionnaires, recording symptoms, and messaging nurses. They considered a 48-hour nurse response time appropriate and found the appointment overview and knowledge base clear and relevant. They found it appropriate to receive blood test and treatment notifications 2 and 5 days before treatment. However, they occasionally experienced issues such as slow performance, freezing, or being logged out, requiring reinstallation (Table 3).

**Table 3.** Examples of feedback from patients testing the prototype.

Prototype testing	Feedback
Patient 1	“Sometimes it is running very slowly or freezing, so I get excluded and must download it again”
Patient 2	“Sometimes I fill in the wrong questionnaire (SCCAI or HBI) because I choose the wrong one”
Patient 3	“It’s easy to message the nurses”

### Final Design

Patients and health care professionals, including those not present at the prototype tests, were asked to provide feedback on the interface, usability, and content (Table 4). On the basis of their input, minor adjustments were made, for example, restricting patients to a single questionnaire (Simple Clinical Colitis Activity Index or Harvey-Bradshaw Index) to avoid completing the incorrect one (Table 3).

**Table 4.** Features in the app based on the identified needs.

Identified needs based on focus groups and workshops	Features	Goal of the features
Easy access when needed	Chat or messaging	To improve access and self-management
SCCAI^a^ or HBI^b^ questionnaires with free-text space as the dialogue tool	Self-initiated PRO^c^ questionnaires specific to SCCAI or HBI with free-text space	To protect privacy and improve shared decision-making
Appointment plan as overview	Overview of appointments	To prevent loss of control
Treatment and blood sample notifications	Treatment and blood sample notifications	To improve adherence
Transparent and trustworthy knowledge on how to live with IBD^d^	Knowledge base containing information on the following: vaccinations, physical activity, medicine, pregnancy and breastfeeding, diet, and traveling with IBD	To improve self-management
Diary and symptom registration	Self-monitoring via diary and symptom registration with the option of sharing the information with health care professionals when needed	To support patients in preparation before a consultation

a SCCAI: Simple Clinical Colitis Activity Index.

b HBI: Harvey-Bradshaw Index.

c PRO: patient-reported outcome.

d IBD: inflammatory bowel disease.

## Discussion

### Principal Findings

We developed a tailored communication app, using participatory design, to support communication between patients with IBD and health care professionals. Crucial to the study methodology were interactive processes and the genuine active participation of end users and the IT designer, which were grounded in mutual learning and collaborative decision-making. The iterative approach of participatory design was essential in developing the app, as it enabled the involved parties to revise the solutions until a suitable intervention was developed, which led to 6 features in the app. Previous studies show that patients with IBD often struggle to access health care professionals [3,9,10], despite this being crucial for self-management [3-7]. Most IBD apps focus on symptom tracking and patient-reported outcomes related to disease activity and information but lack tools to improve communication [22,34]. Patients with IBD highlight that ideal eHealth solutions should enhance communication, with online messaging preferred [10,35]. This study enabled flexible, needs-driven access by incorporating asynchronous messaging for communication between patients and health care professionals.

During development, patients requested urgent messaging with a 15-minute response during flare-ups, but this was not feasible in outpatient settings. Instead, patients and health care professionals agreed on a 48-hour asynchronous response time, with urgent issues handled by phone. This highlights the need to balance patient needs with clinical realities by involving health care professionals in the design process [36].

Patients also requested a “share symptom registration with staff” feature during severe symptoms. Despite initial concerns from health care professionals about workload, the feature was developed. This highlights not only the important democratic aspects of participatory design but also the shift of power dynamics in the participatory design process [25,36,37]. When patients are given a voice in the management of their condition, health care professionals are provided with new insights and understanding, and this mutual understanding may have a significant impact on the acceptability and implementation of the app.

Participatory design often uses tools that combine telling, making, and enacting to encourage participation [25]. In this study, sticky notes and cut-and-paste posters helped participants share ideas for their future communication with health care professionals. To enhance usability, they also chose figures and illustrations for app content and collaborated with a graphic designer to create an animation.

A strength of this study is the use of the existing platform, which is widely used in southern Denmark, secure, integrated with patient records, and GDPR-compliant, thereby supporting transferability. Conducting the study in 2 clinical settings further enhanced this. Relying on existing, supported technologies also shifts the focus toward content development rather than regulatory concerns.

As a future direction in IBD self-management, integrating wearable devices into apps could help detect flares early [38]. Studies suggest that sweat sensors for cytokines may reliably monitor disease activity. Using wearables such as activity trackers or smartwatches to feed data into the app could enhance remote monitoring and empower patients in managing their health [38].

A deeper exploration into the use of mHealth solutions by individuals with IBD could yield valuable insights.

### Limitations

Focus group knowledge depends on participants’ social interaction. Too much homogeneity can limit discussion, while too much heterogeneity may suppress some views [29]. In this study, patients and health care professionals participated in separate groups. This may have reduced interaction but ensured patients speak openly without fear of professional judgment. Furthermore, a limitation of this study was that the concurrent data collection and solution development restricted the opportunity for timely, in-depth data analysis. This may have impacted how emerging insights were incorporated during the design process, potentially affecting the solution’s responsiveness to participant input.

Broad participant involvement is important to gain diverse perspectives that extend beyond one’s own [37]. In our study, user involvement was based on a broad representation of health care professionals, IT consultants, and designers. However, it was challenging to recruit health care professionals, as user involvement is a time-consuming process. Nevertheless, 3 nurses and 1 doctor attended both workshops; the remaining health care professionals joined only one. This may have had a negative impact on the democratic aspect of the development process among health care professionals. However, both professional groups were represented in both workshops.

Patient recruitment also posed challenges. However, 1 man and 1 woman, both with Crohn disease (aged 36‐65 y), participated in both patient workshops. In addition to these 2 patients, only 1 woman wished to participate in prototype testing. She was also diagnosed with Crohn disease and belonged to the same age group (36-65 y). Hence, the results may not reflect the needs of all patients with IBD. However, in this study, they helped to provide insight into the wishes and needs of the patient group in the context of app development.

Finally, it cannot be excluded that additional patient information such as disease duration, social status, and health literacy could have influenced the mHealth development process.

### Conclusions

This study resulted in the design and development of an app for patients with IBD, using participatory design in close collaboration with patients, health care professionals, and an IT designer, emphasizing mutual learning and inclusive decision-making. Thus, the participants had a significant impact on the eHealth solution, as it was tailored to their needs and clinical setting to support communication and access to care. This likely improved the app’s acceptability and potential for implementation.

The iterative participatory design process, with genuine active participation of end users and the IT designer, was essential in developing the app, as it enabled the parties involved to revise the solutions until a suitable intervention was developed. However, full participant testing is anticipated as funding becomes available. Additional studies, both planned and ongoing, will examine the app’s use in the daily clinical management of patients with IBD.

## Supplementary material

10.2196/69093 Checklist 1 COREQ checklist.

## Abbreviations

COREQ: Consolidated Criteria for Reporting Qualitative Studies
GDPR: General Data Protection Regulation
IBD: inflammatory bowel disease
mHealth: mobile health
